# Impulsiveness and executive functions in Parkinson’s disease

**DOI:** 10.1590/1980-57642018dn13-040007

**Published:** 2019

**Authors:** Bruna de Assis Almeida, Amer Cavalheiro Hamdan

**Affiliations:** 1 Universidade Federal do Paraná, Curitiba, PR, Brazil.; 2 PhD, Department of Psychology, Universidade Federal do Paraná, Curitiba, PR, Brazil.

**Keywords:** impulsiveness, executive function, Parkinson disease, cognition, impulsividade, função executiva, doença de Parkinson, cognição

## Abstract

Cognitive functions, such as impulsiveness and executive functions, are often impaired in Parkinson’s disease. Objective: to analyze the relationship between impulsiveness and executive functions (EF) in people with Parkinson’s disease (PD). Methods: a correlation study involving a sample of 50 patients with an established diagnosis of PD aged 40 years or older was conducted using the following instruments: Demographic Questionnaire, Montreal Cognitive Assessment Basic (MOCA-B), Barratt’s Impulsiveness Scale (BIS-11) and Frontal Assessment Battery (FAB). Results: Pearson’s correlation analysis indicated low associations (p<0.05) between the MOCA-B and BIS-11, with a value of -0.11, and between the FAB and BIS-11, with a value of -0.16. A significant correlation between the MOCA-B and FAB was found, with a value of 0.73. Conclusion: this study revealed an association between EF and other cognitive functions, but no association between impulsivity and EF in Parkinson’s disease.

## RESUMO

As funções cognitivas, tais como impulsividade e funções executivas, muitas vezes estão alteradas na doença de Parkinson. Objetivo: analisar a relação entre a impulsividade e as funções executivas (FE) em pessoas com a doença de Parkinson (DP). Métodos: estudo de correlação, com uma amostra de 50 pessoas a partir de 40 anos de idade, com diagnóstico estabelecido da doença de Parkinson utilizando-se os seguintes instrumentos: Questionário Demográfico, Montreal Cognitive Assessment Basic (MOCA-B), Escala de Barrat para Impulsividade (BIS-11) e *Frontal Assessment Batery* (FAB). Resultados: a análise de Correlação de Pearson indicou baixas associações (p<0,05) entre o Moca-B e BIS-11 com -0,11 e o FAB com o BIS-11 tiveram um valor de -0,16. O valor que teve significativa correlação apareceu no Moca-B com o FAB 0,73. Conclusão: este estudo evidenciou uma associação entre as FE e outras funções cognitivas, mas não evidenciou associação entre impulsividade e funções executivas na doença de Parkinson.

Parkinson’s disease (PD) is a degenerative neurological disease that affects people of all ethnicities and economic classes.^1^ It is a progressive, irreversible disease that can debilitate patients in advanced stages, based on the motor symptoms described by the Hoehn and Yahr scale.^2^ In summary, the symptoms of Stage I are unilateral involvement with minimal or no functional impairment; Stage II is defined as bilateral or midline involvement, without impairment of balance; Stage III is initial signs of impaired righting reflexes; Stage IV is fully developed, severely disabling disease; and stage V is confinement to bed or wheelchair.^2^

There is occurrence of neurotransmitter changes, such as degeneration of substantia nigra, which reflects the death of dopaminergic neurons.^3^ The disease is also linked to changes in serotonin, noradrenaline, and acetylcholine in brain function,^3^ and is characterized by three main symptoms: tremor, bradykinesia and rigidity. Postural instability is also very common, and there are other widely shared non-predominant symptoms such as sialorrhea, sweating, increased peripheral vagal tone, blepharospasm, psychiatric and psychotic symptoms, as well as cognitive disorders.^4^^,^ 5

Additionally, according to Diamond (2014, pg. 136): “executive functions (EF)^6^ are essential skills for mental and physical health, school and life success, cognitive, social and psychological development, quality of life, and public safety”.

Currently, impulsivity is considered to have a negative impact on the quality of life of PD patients, whether in academic or work performance, because when behaviors and actions become impulsive this can pose risks that interfere with performance and may often lead to stress.^7^ In other words, impulsiveness and EF are necessary for humans to perform basic tasks related to insertion and adaptation to their environment.

Thus, in light of the physical and cognitive alterations discussed above, the goal of evaluating impulsiveness and EF is justified, since these variables are directly related to the impact of symptoms on individuals with PD. The literature also indicates that 72.5% of people with PD present significantly altered levels of EF and impulsivity.^8^ These findings highlight the influence of EF on people with PD, and indicate the relevance of addressing this issue in this context. 

In order to find papers addressing EF and impulsivity variables in PD, PubMed searches were performed with the words “Parkinson”, “executive functions” and “impulsivity”. This resulted in the retrieval of 25 studies, of which only two showed the two variables acting together, namely: Fonoff et al. (2015) and Leroi et al. (2013). These studies demonstrated differences in EF and impulsivity in individuals with PD.^9^^,^ 10 The findings raise questions regarding the cognitive changes related to impulsivity and brain and chemical function.

In view of the above, the present study aimed to analyze the relationship between impulsiveness and EF in people with PD.

## METHODS

**Participants.** The sample comprised 50 individuals with PD. Demographic characteristics, such as gender, level of education, marital status and years since disease diagnosis were also assessed (see Table 1 in Results). 

**Table 1:** Demographic data for the sample (n=50).

		Mean	Median	SD	Min	Max	Fi	F%
**Age in years**		67.7	67.5	9.91	43	88		
**Sex**	Male						25	50
	Female						25	50
**Educational level**	Middle school						26	52
	High School						10	20
	University						14	28
**Marital status**	Single						4	8
	Married						28	5.6
	Divorced						9	18
	Widowed						9	18
**Years since diagnosis**		9.38	9	5.72	1	25		

SD: standard deviation; MIN: minimum values; MAX: maximum values; Fi: absolute frequency; F%: relative frequency.

Data collection was performed at the Associação dos Portadores de Parkinsonismo do Paraná (Paraná Parkinsonism Patients Association). Inclusion criteria were all subjects over the age of 40 years, with an established diagnosis of PD at mild to moderate stages. Individuals of both sexes and of all educational levels who had the autonomy to answer the questions were eligible. Exclusion criteria: individuals under 40 years of age, diagnosed with any other major neurocognitive disorder and/or who had a history of acquired brain injury, or visual or auditory impairment. Patients at stage IV or V on the Hoehn and Yahr scale were excluded.^2^

### Instruments

The Demographic Questionnaire is a survey that collects the patient’s identification data, including name, sex, age, educational level, medication, and year of disease diagnosis. The instruments were applied by researchers to aid in the assessment of patients.

The Montreal Cognitive Assessment Basic (MOCA-B) is a cognitive screening instrument for fast application^11^ that assesses eight different domains: attention and concentration; executive functions; memory; language; visuoconstructional skills; conceptual thinking; calculations, and orientation. The maximum score is 30 points, the cut-point is 26, and scores below this number indicate alterations; in addition, the test takes an average of 10 minutes to apply. The Portuguese version is considered valid for this study, since authors such as Sarmento (2009) have described it as reliable and sensitive for Brazilian subjects.^12^

The Barratt Impulsiveness Scale (BIS-11) is a scale comprised of 30 items^13^ that evaluates motor, attention, and planning components, which are consecutively characterized by action inhibition, decision-making, and planning understanding. Each item is rated on a scale of up to 4 points: 1 = rarely or never; 2 = from time to time; 3 = frequently and 4 = almost always or always. This scale has been adapted for use into Portuguese.^14^ In a study involving different groups of patients with PD, average score was 58.8 points in individuals with no other disorders, where this parameter was used in the present study, since there are no defined cut-off points.^15^

Finally, the Frontal Assessment Battery (FAB) is a neuropsychological instrument used to evaluate EF.^16^ It is composed of six items: 1-conceptualization; 2-mental flexibility; 3-motor programming; 4-sensitivity to interference; 5-inhibitory control, and 6-environmental autonomy. The FAB detects the functioning of brain regions that are important for the performance of EF and is useful in the differential diagnosis of brain pathologies.^17^ The highest score indicates preservation and has a maximum value of 

18 points, but 12 points can be considered the cut-off point for the population with changes due to PD.^17^ The FAB was adapted to Portuguese.^18^

### Procedure

The research data was collected after the approval of the Ethics Committee of the Hospital de Clínicas of the Federal University of Paraná, under CAAE permit n° 91526718.6.0000.0102 In addition, all subjects who agreed to take part on this study signed an informed consent form. The data collection lasted about forty minutes for each participant.

### Statistical analysis

For the descriptive analysis of the data, the mean, median, standard deviation, minimum/maximum values, and absolute and relative frequency were used. In the inferential analysis, Pearson’s linear correlation coefficient was used with α (*alpha)*<0.05 for null hypothesis rejection. Sample size was calculated using G Power sample size calculation software.^19^ The final sample of 50 participants, when computed for an effect size of 0.4 (medium effect size) and alpha of 0.05, yielded an actual power of 0.84.

## RESULTS

The sample comprised 50 participants and the variables selected were age, sex, education, marital status and years since diagnosis of the disease. The minimum age was 43, maximum age was 88 years and the mean value for years since the diagnosis of Parkinson’s disease was 9.38. In relation to educational level, the majority (28%) of participants had University education. For marital status, 28 (56%) were married and the gender distribution was 50/50.

Table 2 presents the scores on the tests and scales applied in the neuropsychological evaluation. Participant scores were highly variable for all the instruments. The mean scores on the MOCA- B and FAB were below cut-off points, whereas the mean BIS score was higher than expected.

**Table 2:** Raw scores on the scales and tests.

	Mean	Median	SD	Min	Max
MOCA-B	19.3	20	6.18	2	29
FAB	11.6	12	4.43	3	18
BIS-11	64.9	64.5	9.55	44	87

SD: standard deviation; Min: minimum values; Max: maximum values. MOCA-B: Montreal Cognitive Assessment Basic; FAB: Frontal Assessment Battery.

**Table 3:** Pearson's Correlation Analysis.

	FAB	BIS-11
MOCA-B	0.73*	-0.11
FAB		-0.16
BIS-11		

* p<0.001

Pearson’s correlation analysis indicated significant correlation between the Moca-B and the FAB (0.73). However, no significant associations were found between the Moca-B and the BIS-11 (-0.11) or the FAB and the BIS-11 (-0.16).

## DISCUSSION

The main purpose of this article was to analyze the extent of the relationship between impulsiveness and EF in people with PD. The results showed no association between impulsiveness and EF in PD, but revealed a significant correlation between EF and cognition.

Other studies report similar results, where main changes in EF found on neuropsychological tests were observed on the Trail Making A and B, which evaluate cognitive flexibility, as well as on the Go-No-Go task.^20^ In a longitudinal study, some authors also found correlations between tasks related to EF and operation of the frontal lobe in patients matched with controls. However, in a reevaluation, a significant progression of cognitive dysfunction was observed between the two groups measured using the FAB.^20^

Macuglia et al. (2015), found significant differences between PD and non-PD groups regarding the assessment of EF and impulsiveness. Moreover, 72.5% of PD patients presented executive dysfunction. Therefore, the differences were significant regarding perseverance, mental flexibility, attention, working memory, processing speed, inhibition of impulses, and visual perception.

The EF evaluated by the FAB are more closely related to frontal lobe functions^18^ and in the present study were correlated with the Moca-B (0.73), an instrument that assesses several types of cognitive functions.^11^ The above-mentioned studies showed similar findings, serving as a strong indication that some cognitive domains are dependent on the proper functioning of EF^21^ and vice-versa.

On the other hand, the study by Bentivoglio et al. (2013), which investigated the relationship between neuropsychological and behavioral measures associated with the development of Impulse Control Disorders (ICD) in patients with PD, with and without ICD, and without significant cognitive deficits, presented similar results. Although no significant differences between the variables were found, the ICD group had worse performance on neuropsychological tasks sensitive to frontal lobe dysfunction when assessed with the Iowa Gambling Task, a measure of decision-making, and on the Go-No-Go test of the FAB, which evaluates motor impulsivity. The group with ICD, in comparison to the group without ICD, also made more errors related to the inhibition of automatic responses.^15^

Dopamine agonists, that aid the therapeutic efficacy of levodopa, can increase the risk for Impulse-Compulsive Disorders three-fold.^22^ Other studies report the association between impulsive behavior markers and ICD rates, indicating that these individuals have worsening symptoms when dopaminergic agonist is ON.^10^ However, in the present study, no instruments correlated significantly with the BIS-11, which assesses impulsive rates, even with all patients using levodopa for at least one year.

This study had some limitations due to the absence of a control group. However, it contributed to further the knowledge about the relationships between EF, cognition and impulsiveness in PD, since they are interconnected. Finally, although the study contributes to a better understanding of the predisposition for and treatment of EF changes in PD, it reveals gaps in this field. Therefore, future research could focus on the specificities of the instruments that evaluate EF, cognition and impulsiveness in a bid to better understand these tools. This may help to find correlations between cognitive evaluation and EF. Participants could also be stratified into groups according to level of disease progression, among other possible approaches.
